# Cross‐lateralisation in children with attention‐deficit/hyperactivity disorder and motor skill performance

**DOI:** 10.1002/ijop.12658

**Published:** 2020-01-27

**Authors:** Martin Musálek, Sara M. Scharoun Benson, Alena Lejcarova, Pamela J. Bryden

**Affiliations:** ^1^ Faculty of Physical Education and Sport Charles University Prague Czech Republic; ^2^ Department of Kinesiology University of Windsor Windsor Ontario Canada; ^3^ Department of Kinesiology and Physical Education Wilfrid Laurier University Waterloo Ontario Canada

**Keywords:** Attention‐deficit hyperactivity disorder (ADHD), Cross‐laterality, Fine motor, Gross motor, Sex‐related differences

## Abstract

Cross‐lateralisation and increased motor difficulties have been reported in children with attention‐deficit/hyperactivity disorder (ADHD). Nevertheless, the question of how crossed (i.e. mixed preference) or uncrossed (i.e. same side preference) lateralisation impacts motor performance in children with ADHD has yet to be examined. In this study, previously validated observational measures of hand and foot preference were used to identify right‐handed children with ADHD who display cross‐ (*n* = 29) and uncross‐lateralisation (*n* = 31). An uncross‐lateralised typically developing (TD) group (*n* = 32) was also identified, and included as a control. Motor performance was assessed with seven valid and reliable fine and gross motor tasks performed with both preferred and non‐preferred limbs. Group, task and sex‐related effects were examined. Findings revealed that male (but not female) cross‐lateralised children with ADHD performed significantly worse, respectively, in two of the fine motor tasks (spiral tracing [*p* < .01], and dot filling [*p* < .05]). Results suggest that cross‐lateralised hand and foot preference may affect complex motor skills in male children with ADHD. Furthermore, characteristics of ADHD may manifest differently in male and female children. Findings highlight the importance of considering both hand and foot preference when targeting motor interventions for children with ADHD.

Attention‐deficit/hyperactivity disorder (ADHD) is the most commonly diagnosed neurobehavioural disorder (Willcutt, 2012). Although classified by a disruption of inattention and/or hyperactivity‐impulsivity, challenges with fine and gross motor activities are also common (Kaiser, Schoemaker, Albaret, & Geuze, 2015). Characteristics, presence and probability of diagnosis have been linked to cerebral lateralisation of motor function.

Behavioural studies typically use handedness to infer such patterns of lateralisation, yet questions remain regarding the relationship between characteristics of ADHD, lateralisation and motor difficulties. One factor commonly overlooked is the occurrence of cross‐lateralised eye, hand, foot and/or ear preference (e.g. right‐hand and left‐foot, left‐eye and right‐hand etc.). Seldom displayed in typical development, the occurrence of cross hand‐eye lateralisation is more prevalent in children with developmental learning and reading challenges, and ambiguous lower limb preference has been reported (Connolly, 1983). As ADHD is related to both atypical brain lateralisation (Hale et al., 2014) and altered interhemispheric connectivity (Gilliam et al., 2011), it has been argued that ADHD may be categorised by “a general state of anomalous lateralization” (Reid & Norvilitis, 2000, p. 314).

Recent research has demonstrated left‐handers are more likely to have ADHD than right handers (Simões, Carvalho, & Schmidt, 2017). Furthermore, associations between non‐right and/or mixed‐handedness have been linked to some, but not all characteristics of ADHD (Rodriguez et al., 2010; Schmidt, Carvalho, & Simoes, 2017). Rodriguez et al. (2010) found mixed handedness was associated with inattention, but not hyperactivity. Likewise, Schmidt et al. (2017) indicated impulsivity may be dependent on direction and consistency of handedness. Lin and Tsuang (2018) also found a significant relationship between mixed handedness and inattention; however, the difference in hyperactivity and impulsivity scores did not differ. Others (e.g. Ghanizadeh, 2013) have noted no difference in handedness between children with ADHD and typically developing (TD) peers.

Although most work has focused on measuring upper limb function in ADHD, research assessing lower limb performance has also been conducted. For example, recent work from Tran and Voracek (2018) revealed the probability of ADHD was one and half times greater in adults with mixed‐ compared to consistent‐footedness. Higher inattention and impulsivity scores were also associated with left‐ and mixed‐footedness.

The current study considered both handedness and footedness, building upon Scharoun, Bryden, Otipkova, Musalek, and Lejcarova (2013) who assessed fine and gross motor skills in 9‐ to 11‐year olds with ADHD and their TD peers. Children performed seven tasks with the preferred and non‐preferred limbs (i.e. hands or feet). Direction of upper and lower limb preference were the same. Findings revealed children with ADHD displayed poorer performance in more complex motor tasks. No differences emerged in less complex motor tasks, attributed to the focus on gross motor speed, as opposed to the complex limb coordination required in other tasks (e.g. Meyer & Sagvolden, 2006).

Using the same fine and gross motor tasks as Scharoun et al. (2013), the goal of the current study was to assess motor performance in children with ADHD who display both crossed/uncrossed hand‐foot preference, in addition to a control group of typically developing children with uncrossed preference. Due to the low prevalence of crossed lateralisation in typically developing children, it was not feasible to include this comparison group. We hypothesised that cross‐lateralised children with ADHD would perform significantly worse in fine and gross motor tasks compared to the other sub‐groups.

A secondary aim was to assess sex‐related differences. While it is generally reported that males are more likely to be left‐handed, sex differences in brain structure and function underlying language processes have also been revealed (e.g. Sommer, Aleman, Somers, Boks, & Kahn, 2008). Sex is also a significant moderating factor in the assessment of ADHD. A meta‐analysis (Hasson & Fine, 2012) revealed the difference among boys with and without ADHD was significantly larger than the difference among girls with and without ADHD. Scharoun et al. (2013) only revealed differences in male and female children with ADHD in one fine motor task. Here, male children with ADHD were faster with the non‐preferred hand, whereas female children with ADHD were faster with the preferred hand. As such, we did not anticipate significant differences in the performance of male and female children, other than those which were revealed previously.

## METHODS

### Participants

This study included 92 children ages 9‐ to 11‐year olds (60 with ADHD; 31 female, 29 male; 32 typically developing; 16 female, 16 male) from the Czech Republic (*M*_age_ = 10.2, *SD* = .76). Children with ADHD were recruited from elementary schools for children with specific learning disorders identified by the National Institute for Pedagogy and Psychology. Children were diagnosed at 5–6 years. As per Czech legislation, assessment is repeated annually using standard tools: the Statistical Classification of Diseases and Related Health Problems (known in Czech as MKN‐10; World Health Organization, 1992), the Wechsler Intelligence Scale for Children (WISC‐III; Krejčířová, Boschek, & Dan, 2002) and the Strengths and Difficulties Questionnaire (Goodman, 1997). To ensure sensitive information remained confidential, researchers were not privy to children's personal files; therefore, use of this information, including identification of ADHD sub‐types, was not feasible. Typically developing children were recruited from a general elementary school that was randomly selected from a reference list of schools in Prague. The Ethics Committee of the Faculty of Physical Education and Sport at Charles University approved the research. Parents of all participants signed an informed consent form. All procedures performed in studies involving human participants were in accordance with the ethical standards of the institutional research committee and with the 1964 Helsinki Declaration and its later amendments or comparable ethical standards.

### Apparatus and procedures

To ensure consistency in study procedures, one researcher completed all data collection and scored all measures. To identify whether children displayed crossed or uncrossed‐lateralisation, previously validated observational measures of hand and foot preference were used (Musálek, 2013). Children performed three trials of each of the tasks, one‐on‐one with one researcher. Hand preference tasks included: (a) throwing a ball at a target, (b) ringing a bell and (c) cutting with a (child‐safe) knife. Foot preference tasks included: (a) writing a letter “T” on the floor with one foot and (b) kicking a ball. Limb choice was recorded in each of the aforementioned tasks. Only those who completed all three hand preference tasks with the right hand were included in analyses. This resulted in three groups: (1) Cross‐lateralised children with ADHD (i.e. right‐handed and left‐footed; *n* = 14 male, *n* = 15 female); (2) uncross‐lateralised children with ADHD (i.e. right‐handed and right‐footed; *n* = 15 male, *n* = 16 female) and (3) uncross‐lateralised typically developing control group, (i.e. right‐handed and right‐footed; *n* = 16 male, *n* = 16 female).

As this study intended to extend the work of Scharoun et al. (2013), the same seven valid and reliable motor tasks were used to assess motor performance (Musálek, 2013). This included three fine motor skills: (1) spiral tracing, (2) dot filling and (3) tweezers and beads; and four gross motor skills: (1) small plate tapping, (2) large plate tapping, (3) twist box and (4) foot tapping. Participants completed the tasks at school, outside their classroom in a designated room. An employee at the school was present at all times. Data were collected by one researcher on two subsequent mornings, at the same time, in attempt to limit possible changes in performance caused by fatigue. All participants executed tasks in the same order. On Day 1, spiral tracing, foot tapping, tweezers and beads and large plate tapping were completed. On Day 2, dot filling, small plate tapping and twist box were completed. Participants completed each task with the preferred hand/ft, followed by the non‐preferred hand/ft. All upper limb tasks were performed while seated at a table.

### Spiral tracing

A paper and pencil task, participants traced between lines of a spiral as quickly and accurately as possible. The largest diameter of the spiral was 41 mm, and the width was 2 mm. Participants were not permitted to reposition the sheet. Drawing outside the lines and/or touching the lines was deemed an error and penalised by 2 seconds. Time to completion (i.e. the moment the participant's pen crossed the finish line) was recorded for separate trials with preferred and non‐preferred hands.

### Dot filling

Also a paper and pencil task, participants were presented with a sheet of 90 identical circles (2 mm diameter) and asked to place a dot in as many circles as possible within 30 seconds. Separate preferred and non‐preferred hand trials were performed. Only dots placed within the circles were counted.

### Tweezers and beads

This task included two open matchboxes, placed one behind the other (i.e. one closer to the participant, one further away), and a pair of tweezers placed on the desk 150 mm in front of the participant. The matchbox closer to the participant was filled with 20 beads (5 mm in diameter), and the second was empty. Participants were instructed to use the tweezers to move as many beads as possible (one by one) from the full box to the empty box. The number of beads transported in 30 seconds was recorded for separate preferred and non‐preferred hand trials.

### Twist box

This task included a closed matchbox placed on the table in front of the participant at the midline. Starting in a pronated position and holding the matchbox between the thumb and index finger, the task involved using one hand to rotate the box 180°. The box had to touch the table at all times; therefore, it could not be lifted. Each trial (preferred and non‐preferred) took 30 seconds. Performance was video recorded and coded offline using Dart fish (program version 4.5.2.0.; Dartfish HQ, Fribourg, Switzerland). Each correct 180° rotation (i.e. twist) was recorded.

### Small plate tapping

The Lafayette Tapping Board Test (Lafayette Instruments Co.) was used. The electronic plate (30 cm in length) with two metal surfaces was placed at the participant's midline. The electronic plate was connected to an electronic meter through a cable. A metal tool, connected to the plate with a cable, was used for tapping with one hand. The participant was asked to tap the metal surfaces of the plate with the metal tool for 30 seconds (separate preferred and non‐preferred hand trials). While the number of taps was recorded by the instrument on a digital display, the display was not shown to participants, to prevent any undue influence on performance.

### Large plate tapping

The task included two square targets (15 cm sides; labelled “right” and “left”) attached to a Table 70 cm apart from each other and a middle target (15 cm side) with a picture of a hand in the middle. Starting with the designated hand on the middle target, participants were required to tap the targets, when signalled, alternating between left and right. Each trial took 30 seconds (preferred and non‐preferred hand trials). Performance was video recorded and coded offline using Dart fish (program version 4.5.2.0.; Dartfish HQ, Fribourg, Switzerland). The number of taps was recorded.

### Foot tapping

This task required the participant to stand next to a table with his/her preferred leg adjacent to the table. Subsequently, the participant tapped his/her foot in front of and behind the standing leg, alternating between heel and toe for 30 seconds (preferred and non‐preferred foot trials). Performance was video recorded and coded offline using Dart fish (program version 4.5.2.0.; Dartfish HQ, Fribourg, Switzerland). The number of taps was recorded.

### Data analysis

Data normality (Shapiro–Wilk, Anderson‐Darling, Kolmogorov–Smirnov), circularity and covariance matrices equal (Box‐M test) were verified. Data were then entered into a three‐way repeated measures analysis of variance (ANOVA). The within‐subjects factor was the limb used to complete the task (non‐preferred, preferred). Between‐subjects factors included group (cross‐lateralised children with ADHD, uncross‐lateralised children with ADHD, uncross‐lateralised TD children) and sex (males, females). Subsequently, data were analysed using separate one‐way analyses of variance (ANOVAs) that considered the hand used to complete the task and sex. Statistical significance was set at *p* < .05. Only results with large effect sizes (Hays (*ω* ^2^ > .14) were considered (Olejnik & Algina, 2003).

## RESULTS

Assumptions for ANOVA were verified in exploratory data analysis (EDA). In four of the seven tests (Dot filling, Tweezers and beads, Small plate tapping and Foot tapping) data normality was confirmed. In Spiral tracing, Twisting box and Large plate tapping, normality was rejected in at least one test (Table 1); however, covariance matrices were equal and circularity was confirmed in all tests (Table 2). Due to certain robusticity of ANOVA against violating the normal distribution of data (Blanca, Alarcón, Arnau, Bono, & Bendayan, 2017Schmider, Ziegler, Danay, Beyer, & Bühner, 2010) approaches along with using practical significance coefficient Hays 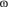 ^2^, we used repeated measures ANOVA as well as separate one‐way ANOVAs.

**Table 1:** Data normality considering extremity preference and diagnoses

Tests	Shapiro‐Wilcox (range)	Anderson‐Darling (range)	Kolmogorov–Smirnov (range) test criterion 0.16
Spiral tracing	0.063–0.41	0.057–0.35	0.08–0.18*
Dot filling	0.08–0.54	0.19–0.57	0.10–0.14
Tweezers and beads	0.065–0.80	0.06–0.53	0.10–0.13
Twist box	0.02*–0.50	0.03*–0.50	0.08–0.15
Small tap test	0.16–0.82	0.09–0.85	0.07–0.11
Large tap test	0.02*–0.25	0.12–0.55	0.10–0.18*
Foot tapping	0.14–0.88	0.10–0.72	0.06–0.15

* Not normally distributed data on *p* < .05.

**Table 2:** Covariance matrices equal and circularity

Tests	Box's M	*F*‐value	Probability level
Spiral tracing	8.59	1.39	0.22
Dot filling	10.74	1.73	0.13
Tweezers and beads	6.78	1.09	0.36
Twist box	4.21	0.68	0.66
Small tap test	2.13	0.34	0.91
Large tap test	8.02	1.29	0.26
Foot tapping	11.18	2.06	0.11

Overall, children performed significantly better with the preferred hand/foot compared to the non‐preferred hand/foot. With the exception of twist box, children with ADHD performed all tasks significantly worse than TD children. Moreover, spiral tracing, twist box and small plate tapping revealed significant differences between females and males. Results from each task are described in detail below.

### Spiral tracing

Significant main effects of group, sex and limb were revealed. Children with ADHD performed significantly worse than their TD peers, *F*(2, 91) = 15.19; MSE = 902.29, *p* < .01, Hays *ω* ^2^ = .06. Furthermore, females ($\bar{x}=42.9\pm 10.43)$ were faster than males ($\bar{x}=46.6\pm 13.7)$ *F*(1, 92) = 10.92, MSE = 648.64, *p* < .01, Hays *ω* ^2^ = .07. Finally, performance with the preferred hand ($\bar{x}=35.23\pm 5.54$) was faster than non‐preferred ($\bar{x}=54.46\pm 9.55$) *F*(1, 185) = 504.48, MSE = 16,758, *p* < .001, Hays *ω* ^2^ = .28.

**Figure 1:**
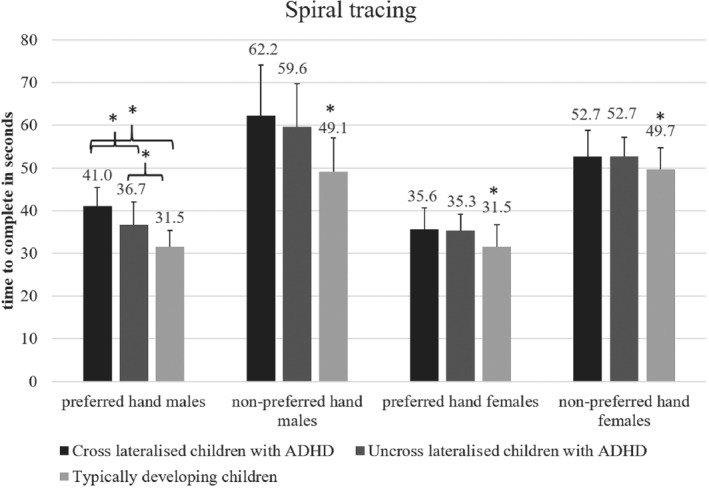
Spiral tracing revealed poorer performance in children with ADHD, particularly in cross‐lateralised males with ADHD.

The separate one‐way ANOVA revealed that cross‐lateralised males with ADHD took significantly longer ($\bar{x}=41\pm 4.42)$ with the preferred hand than uncross‐lateralised males with ADHD ($\bar{x}=36.7\pm 5.32)$ and typically developing males ($\bar{x}=31.5\pm 3.77),$ *F*(2, 47) = 16.00, MSE = 340.21, *p* < .01, Hays *ω* ^2^ = .39. On the other hand, cross‐lateralised females with ADHD did not perform differently than uncross‐lateralised females with ADHD (Figure 1).

### Dot filling

**Figure 2:**
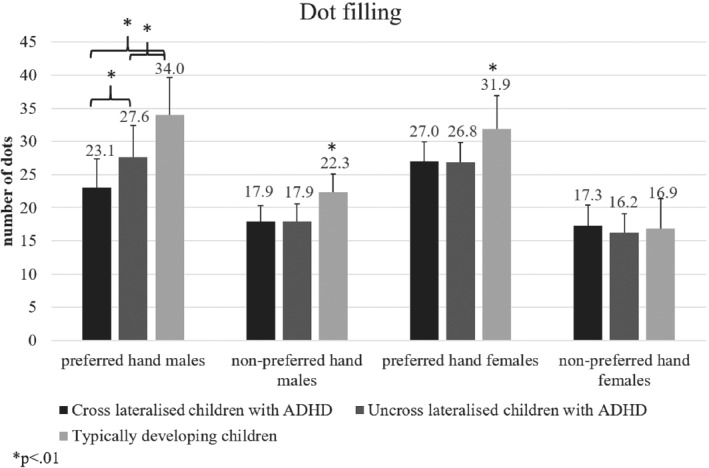
Children with ADHD made fewer dots than typically developing children and cross‐lateralised males with ADHD made fewer dots with the preferred hand.

Children with ADHD made significantly fewer dots, *F*(2, 184) = 15.79, MSE = 169.51, *p* < .01, Hays *ω* ^2^ = .08, compared to TD peers. Males and females did not differ (*p* > .05). All participants made significantly more dots with the preferred hand *F*(1, 185) = 465.86, MSE = 6528.92, *p* < .001, Hays *ω* ^2^ = .23, $(\bar{x}=28.5\pm 5.48$) compared to the non‐preferred hand ($\bar{x}=16.5\pm 3.31$). A significant interaction was found between group and limb, *F* (2, 184) = 15.86, MSE = 169.51, *p* < .01, Hays *ω* ^2^ = .09; Figure 2. Cross‐lateralised children with ADHD displayed significantly smaller difference in performance between the preferred and non‐preferred hands compared to TD children.

Results from separate ANOVAs that included sex as a factor revealed that cross‐lateralised males with ADHD made significantly fewer dots with the preferred hand ($\bar{x}=23.07\pm 4.31$) compared to uncross‐lateralised males with ADHD ($\bar{x}=27.66\pm 4.77$) and TD males ($\bar{x}=34.1\pm 5.66$; *F*(2, 47) = 22.87, MSE = 483.72, *p* < .01, Hays ω^2^ = .48). Further, no significant difference was revealed between females with and without ADHD in non‐preferred hand performance (*p* > .05).

### Tweezers and beads

**Figure 3:**
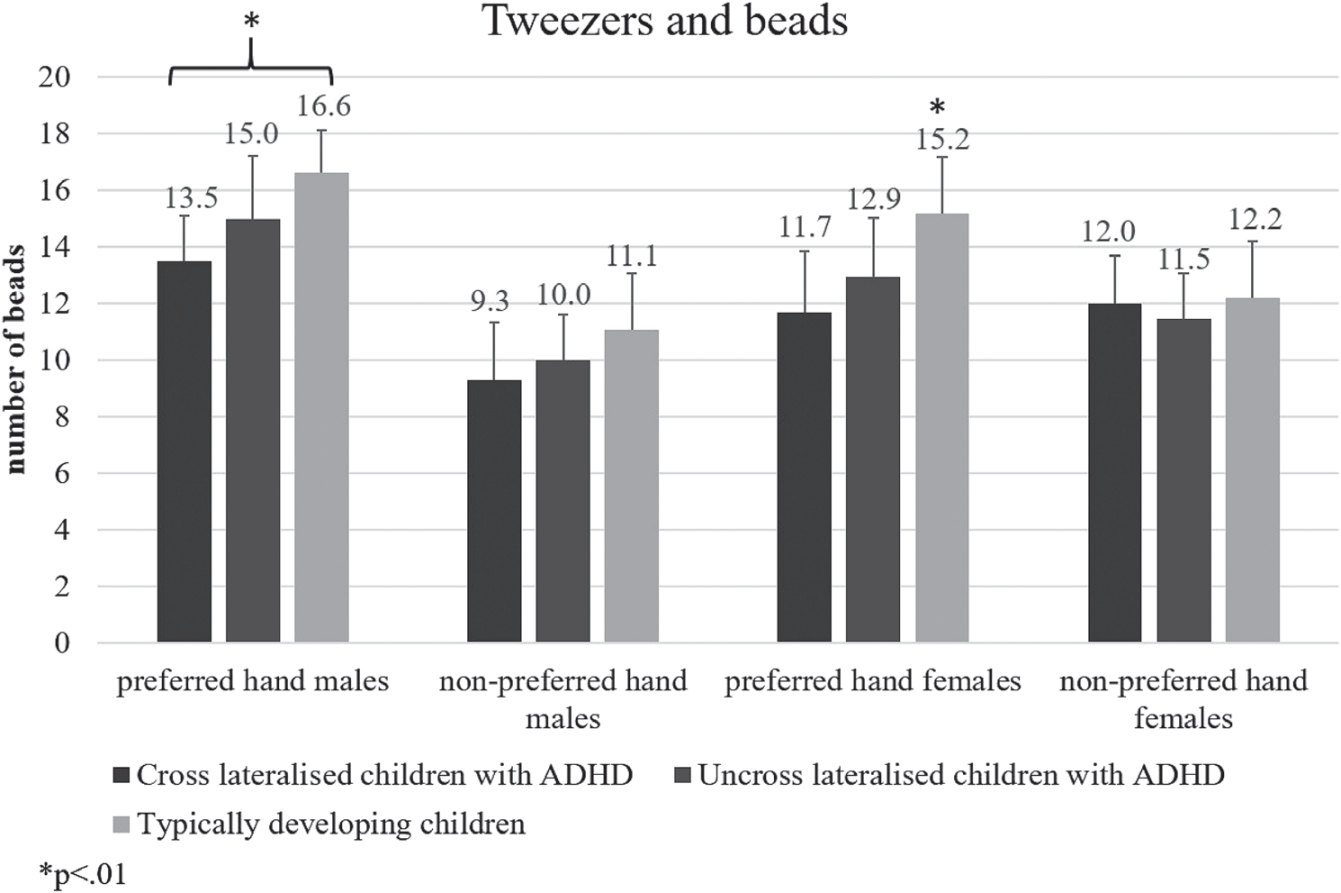
Children with ADHD—cross‐lateralised males in particular—moved significantly fewer beads with the preferred hand compared to typically developing children.

Main effects of group and sex were not significant (*p* > .05). Nevertheless, the main effect of limb revealed all participants moved significantly more beads with the preferred hand, *F*(1, 185) = 153.17, MSE = 376.14, *p* < .001, Hays *ω* ^2^ = .42; $\bar{x}=14.3\pm 2.54,$ compared to the non‐preferred hand ($\bar{x}=11.3\pm 2.3)$. A significant interaction between limb and sex, *F*(2, 184) = 79.95, MSE = 196.33, *p* < .001, Hays *ω* ^2^ = .24, revealed a significant difference in preferred and non‐preferred hand performance exclusive to male participants. The interaction between group and limb revealed children with ADHD moved significantly fewer beads compared to their TD counterparts, *F*(2, 91) = 22.82, MSE = 107.88, *p* < .01, Hays *ω* ^2^ = .14, when using the preferred hand. The three way interaction between group, sex and limb, *F*(2, 91) = 9.50, MSE = 36.61, *p* < .01, Hays *ω* ^2^ = .31; Figure 3, further revealed that cross‐lateralised males with ADHD ($\bar{x}=13.5\pm 2.1)$ moved fewer beads with the preferred hand compared to TD males ($\bar{x}=16.6\pm 1.54).$

Results from separate ANOVAs revealed that cross‐lateralised children with ADHD displayed a significantly smaller difference in performance between the preferred and non‐preferred hands, *F*(2, 184) = 3.61; MSE = 8.85, *p* = .034, Hays *ω* ^2^ = .05. However, subsequent power analysis revealed a low level of task power (65%), and thus, significance of the observed difference between average performances of the participants, was insufficient.

### Twist box

No significant differences between children with ADHD and their TD peers emerged. It was observed that females scored significantly worse ($\bar{x}=34\pm 6.1)$ than males ($\bar{x}=38.2\pm 6.5;$ *F*(2, 91) = 18.41, MSE = 508.71, *p* < .01, Hays *ω* ^2^ = .15). Moreover, a significant effect of limb, *F*(1, 185) = 39.39, MSE = 575.50, *p* < .001, Hays *ω* ^2^ = .21, revealed the box was twisted significantly more times with the preferred hand ($\bar{x}=39.7\pm 6.3)$ than the non‐preferred hand ($\bar{x}=36.1\pm 4.9)$.

### Small plate tapping

**Figure 4:**
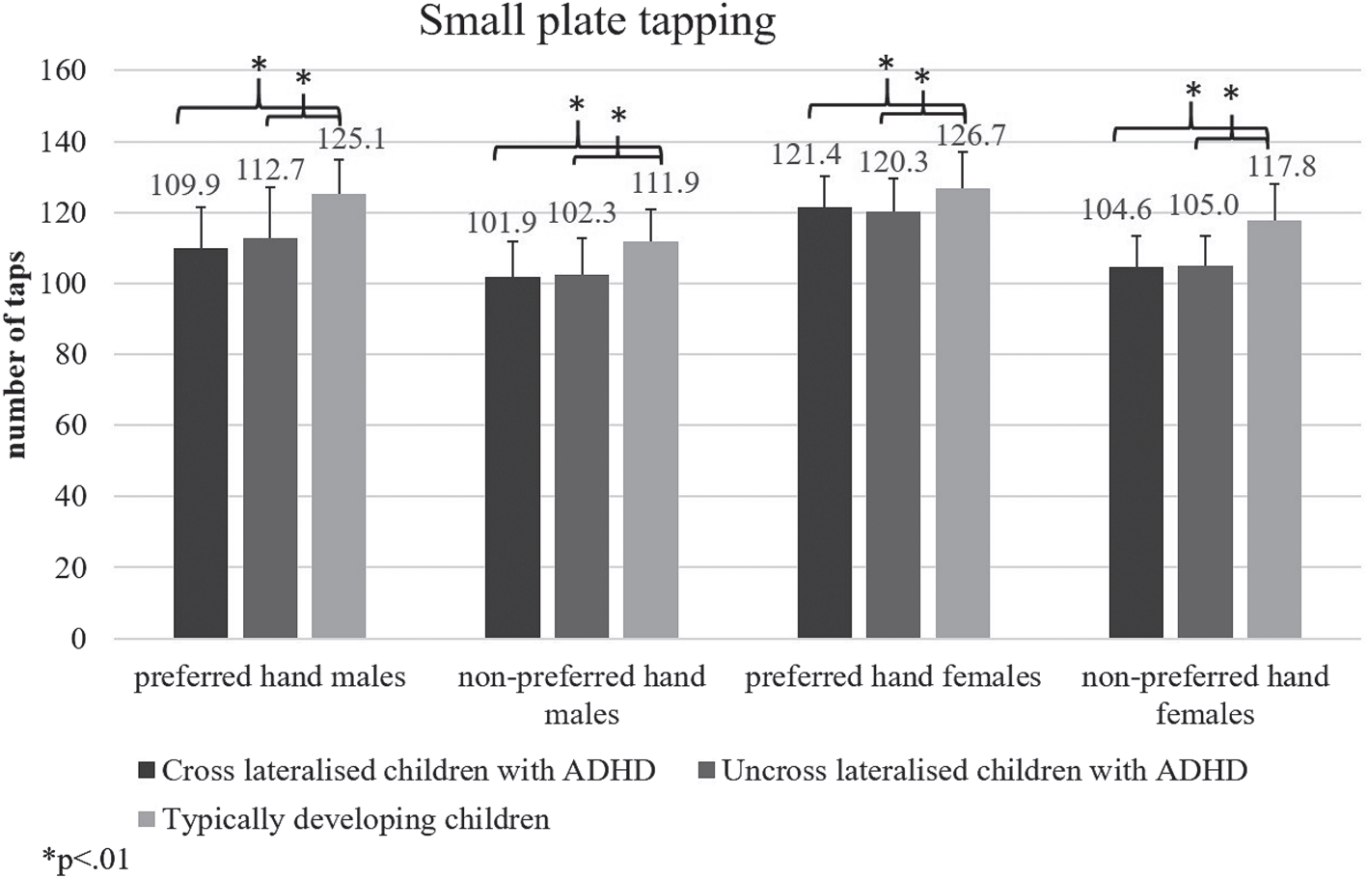
Children wtih ADHD made significantly fewer taps than typically developing peers.

The effect of group was non‐significant; overall performance of children with ADHD and TD peers did not differ in small plate tapping (*p* > .05). Females scored significantly better with the preferred hand compared to males, *F*(2, 91) = 13.16, MSE = 1308, *p* < .01, Hays *ω* ^2^ = .14. A significant difference, *F*(1, 185) = 59.17, MSE = 6731.83, *p* < .001, Hays *ω* ^2^ = .20, was observed in the number of taps completed with the preferred ($\bar{x}=119.2\pm 12.5$) and non‐preferred hand ($\bar{x}=107\pm 10.9$). Further, children with ADHD scored significantly worse than TD peers with both the preferred, *F*(2, 91) = 8.08, MSE = 1048, *p* < .01, and non‐preferred hand, *F*(2, 91) = 14.8, MSE = 1336.46, *p* < .01; Figure 4.

### Large plate tapping

No significant differences were found as a function of group or sex. A main effect of limb, *F*(1, 185) = 8.42, MSE = 284.29, *p* < .01, revealed a significant difference in preferred ($\bar{x}=64.2\pm 6.3$) and non‐preferred hand ($\bar{x}=61.8\pm 5.4$) performance; however, this difference had a low effect size (Hays *ω* ^2^ = .04).

### Foot tapping

**Figure 5:**
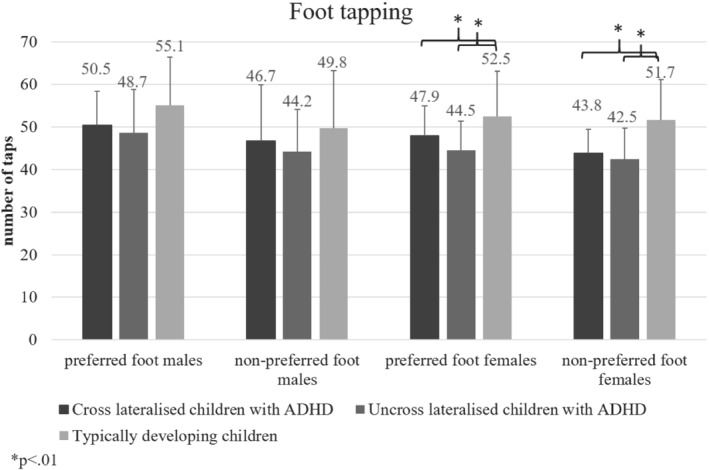
Females with ADHD performed significantly fewer taps than typically developing peers in the foot tapping task.

Effects of group and sex were non‐significant (*p* > .05). For all participants, the preferred foot ($\bar{x}=50\pm 9.07$) performed better than the non‐preferred one ($\bar{x}=46.5\pm 8.2);$ *F*(1, 185) = 15.23, MSE = 533.77, *p* < .01, Hays *ω* ^2^ = .15. Uncross‐lateralised children with ADHD performed significantly worse with the preferred, *F*(2, 91) = 4.91, MSE = 412.58, *p* < .01, Hays *ω* ^2^ = .07, and non‐preferred foot *F*(2, 91) = 4.38, MSE = 45.26, *p* < .05, Hays *ω* ^2^ = .08, than typically developing children. When performing with the preferred foot, females with ADHD (cross‐lateralised: $\bar{x}=47.9\pm 7.1$; uncross‐lateralised: $\bar{x}=44.5\pm 6.7$ performed significantly worse than their TD counterparts ($\bar{x}=52.5\pm 10.6$; *F*(2, 91) = 3.55, MSE = 245.98, *p* < .05; Hays *ω* ^2^ = .11). Likewise, greater performance differences were found in non‐preferred foot performance when comparing TD females ($\bar{x}=51.7\pm 9.5)$ to cross‐ ($\bar{x}=43.8\pm 7.1)$ and uncross‐ ($\bar{x}=42.5\pm 7.2)$ lateralised females with ADHD, *F*(2, 91) = 6.30, MSE = 370.88, *p* < .01, Hays *ω* ^2^ = .19. No significant differences were observed in males (Figure 5).

## DISCUSSION

The current study assessed fine and gross motor skills in three groups: (1) cross‐lateralised children with ADHD; (2) uncross‐lateralised children with ADHDand (3) an uncross‐lateralised TD control group. Based on findings from Scharoun et al. (2013), we did not anticipate differences in performance of male and female children. Nevertheless, females performed better than males in spiral tracing overall and better with their preferred hand in small plate tapping. Male children performed the twist box task better than their female counterparts.

We did, however, expect children with ADHD, and, cross‐lateralised children with ADHD specifically, would display less proficient motor skills. Findings offer partial support to our hypotheses. Children with ADHD displayed worse performance than TD peers on all tasks, except twist box, and cross‐lateralisation also had a significant impact on performance. Findings were more robust in male cross‐lateralised children with ADHD in two fine motor tasks (spiral tracing and dot filling), providing support for the notion that ADHD may manifest differently in male and female children. Findings will be discussed in the context of task‐ and sex‐related effects, with the discussion of differences between the limbs embedded within each section, where appropriate.

### Task‐related effects

In line with previous research (Scharoun et al., 2013), children with ADHD performed worse than the typically developing control group in fine motor tasks, as well as in some gross motor tasks. The most robust effects were revealed in spiral tracing and dot filling, and, particularly for cross‐lateralised children with ADHD. When performing with the preferred hand, cross‐lateralised children with ADHD performed worse than both uncross‐lateralised children with ADHD and TD children. These tasks both require a stylus (i.e. writing utensil) to compete, and were the most complex as they require the greatest fine motor control. In the tweezer and beads task, where both proximal and distal upper limb control was required, only cross‐lateralised children with ADHD scored significantly worse than TD children, particularly when performing with the preferred hand. Surprisingly, the performance of uncross‐lateralised children with ADHD only differed from typically developing peers in non‐preferred hand performance within this task. These results are generally consistent with previous work (Scharoun et al., 2013) which revealed that the more complex the motor tasks are, the greater challenges for children with ADHD. Extending the previous work, findings support the notion that that fine motor skills of children with ADHD are affected differently based on pattern of lateralised preference, where children with cross‐lateralised preference have greater challenges. Future work would benefit from assessing how the severity of symptoms relates with strength of cross‐ and uncross‐lateralised preference to further disentangle our understanding of ADHD and motor skill performance.

Findings from gross motor tasks also revealed differences in children with ADHD and TD controls, albeit not to the same extent as was displayed in fine motor tasks. Like Scharoun et al. (2013), no difference emerged in the twist box task; however, in contrast, it was revealed that children with ADHD performed more poorly than typically developing peers in the small plate tapping task. Although a difference also emerged in large plate tapping, *post‐hoc* power was low. Taken together, results from fine and gross motor assessments are concurrent with many previous studies (Scharoun et al., 2013), which have revealed the greatest differences in more complex tasks.

Foot tapping was the only task used to assess lower limb performance. It has been argued that lower limb tasks may be more suitable than upper limb tasks to establish a measure of lateralisation without the confounding effects of social influence, and to assess motor deficiencies (Peters, 1990). Nevertheless, there exists a dearth of literature in this area. Previous work assessing lower limb motor performance in children with ADHD have focused primarily on balance (e.g. Chen et al., 2012). Here, children with ADHD are typically reported to have greater challenges with balance than their TD peers; however, Schlee, Neubert, Worenz, and Milani (2012) observed no difference in static balance. Results from the current investigation revealed all children performed significantly better with the preferred leg. In line with Scharoun et al. (2013), children with ADHD generally scored worse; however, the difference was not statistically significant.

### Sex‐related effects

Overall, we observed less proficient motor performance in males with ADHD compared to their typically developing peers. Nevertheless, in foot tapping, female children with ADHD made significantly fewer taps with both the preferred and non‐preferred leg than their TD peers. There is disagreement in the literature regarding the manifestation of ADHD in male and female children (Nøvik et al., 2006). Evidence supports the notion (e.g. Cole, Mostofsky, Larson, Denckla, & Mahone, 2008) that there are distinct differences, whereby motor control is less impaired in females with ADHD. Sex‐related differences have been attributed to differences in neural maturation (e.g. Onnink et al., 2014) and symptom manifestation (e.g. Biederman et al., 2002). It has been argued that, unlike male children with ADHD who typically present with hyperactive and aggressive symptoms, female children may be up to twice as likely to present with the inattentive form of ADHD, and thus suffer from more internalising symptoms and inattention (Biederman et al., 2002). ADHD thus manifests differently in male and female children, both when looking at behavioural characteristics and motor skill performance.

## CONCLUSIONS

Taken together, findings revealed children with ADHD—those with cross‐laterality in particular—performed the worst in highly complex tasks: spiral tracing and dot filling. It can thus be argued that cross‐lateralisation does play a role in the motor skills of children with ADHD. These findings are concurrent with existing evidence that deficiency in higher‐order cognitive processing underlies motor deficits (e.g. Leung & Connolly, 1998). It is interesting to note that male children with ADHD displayed the most challenges with upper limb tasks; whereby female children with ADHD were least proficient in lower limb tasks. Findings contrast results of Meyer and Sagvolden (2006), who observed worse performance in females with ADHD females. Unfortunately, the majority of research assessing motor skills in children with ADHD has focused on males. As a result, continued research is needed in this area to clarify whether there is a link between the higher prevalence of ADHD in males and subsequent differences in motor performance between the sexes.

### Limitations

A fundamental limitation of the current study is the lack of typically‐developing children with cross lateralised preference. Furthermore, we were unable to obtain data from the Wechsler Intelligence Scale for Children, Strengths and Difficulties Questionnaire, or information on the specific type of ADHD children had been diagnosed with, as per Czech legislation. Three different subgroups of ADHD have been identified, based on differences in diagnostic criteria: ADHD predominantly inattentive (ADHD‐I), ADHD predominantly hyperactive–impulsive (ADHD‐H) and ADHD combined type (ADHD‐C). A systematic review from Kaiser et al. (2015) revealed children with ADHD‐I and ADHD‐C typically display greater challenges with motor skills than children with ADHD‐H. Future would indeed benefit from the assessment of motor skills in cross‐ and uncrossed‐lateralised male and female children within each of the different subgroups of ADHD, while also considering other standardised measures, such as the Wechsler Intelligence Scale.
